# Air Quality in Dental Care Facilities: Update to Current Management and Control Strategies Implementing New Technologies: A Comprehensive Review

**DOI:** 10.3390/vaccines10060847

**Published:** 2022-05-26

**Authors:** Ioannis Tzoutzas, Ioannis Karoussis, Helena C. Maltezou

**Affiliations:** 1 School of Dentistry, National and Kapodistrian University of Athens, 11527 Athens, Greece; tzoudent@dent.uoa.gr (I.T.); ikaroussis@dent.uoa.gr (I.K.); 2 Directorate of Research, Studies and Documentation, National Public Health Organization, 15123 Athens, Greece

**Keywords:** indoor air quality, air splatter, microdroplets, SARS-CoV-2, dental care facilities

## Abstract

The quality of indoor air in healthcare facilities, with an emphasis on dental offices, attracted the attention of the scientific community in the late 1960s. Since then, it has become evident that the indoor air quality is critical in modern dental care facilities for limiting the spread of airborne infections, including vaccine-preventable diseases, and a key component of safety for healthcare personnel and patients. In the past decades, the role of indoor air quality has also been recognized in non-healthcare facilities, given the increasing time spent indoors by humans. During the provision of dental care services, mainly in the field of restorative dentistry, high-speed dental handpieces emitting air and water are used, producing large quantities of aerosol and hovering inside the operations area. In modern dental offices, new devices emitting air/powder for cavities improvement and cleaning as well as for periodontal prophylactic cleaning and aesthetics are used. In addition, a new therapeutic protocol for the removal of bacterial biofilm, targeting treatment for peri-implant diseases and conditions using air-abrasive decontamination technology, has been introduced in daily dental practice. The aim of this non-systemic review is to present the current state of knowledge on the nature and dynamics of air splatters and to provide an update to management and control strategies in dental care facilities, focusing on air purification and ultraviolet devices proposed and used. The findings arising from the limited number of related published articles documenting the reduction in levels of particular matter 2.5 (PM_2.5_), PM_10_ and volatile organic compounds, allow us to conclude that the continuous operation of air purifiers during and after treatment, contributes considerably to the improvement of the indoor air quality in dental care facilities. Moreover, the utilization of air purifiers is highly recommended in dental practice to mitigate spread of infections, including vaccine-preventable diseases. Frequent cleaning and maintenance of the purifier sieves and filters and frequent renovation of the indoor air through physical ventilation by mean of open windows is imperative. More research on environmental contamination and particularly on viral contamination under real dental care conditions is needed.

## 1. Introduction

The emergence of severe acute respiratory syndrome coronavirus 2 (SARS-CoV-2) in late 2019 and the evolution of the coronavirus disease 2019 (COVID-19) pandemic has resulted in major uncertainties in health sciences regarding the precise mechanisms of virus transmission [1,2]. Airborne transmission of SARS-CoV-2 has been implicated in several COVID-19 outbreaks, including an outbreak in a healthcare facility involving a superspreading event [3,4]. Therefore, the identification of the most effective methods and best practices for the protection of healthcare personnel and patients in healthcare facilities constituted a key element of COVID-19 pandemic response plans [1,2]. However, the issue of indoor air quality in healthcare facilities has attracted the attention of the scientific community since the late 1960s [5]. At that time, emphasis was placed on dental offices given that high speed air/water handpieces became drastically popularized in restorative dentistry and polymeric materials prevailed in aesthetic repairs, whereas various essential oils were incorporated in tuples of clinical applications [6]. In recent years, it has become evident that low air quality in healthcare settings in the context of increased work pressure, such as in operating rooms, where the chemical burden and organic contaminators are frequently combined, may be detrimental for both healthcare personnel and patients [7,8]. In particular, building construction and pollutants entering from outdoors may render in-hospital air quality inefficient, mainly because hospitals in urban areas are surrounded by pollutant sources, including automobiles, industries, and central heating systems. Additionally, multiple traditional or innovative treatments and invasive operations may produce microdroplets, steams, and smokes, whose potential hazardous consequences on healthcare personnel has not been sufficiently studied, and are collectively referred to as Newly Identified Health Risks [9]. Consequently, adjusted ventilation systems are imperative in healthcare settings such as dental clinics, laboratories, and operation rooms. Currently, there are gaps in the literature regarding the dynamics of air splatters and in particular in the management and control strategies in dental care facilities, with an emphasis on the operation of air purification and ultraviolet (UV) devises. Furthermore, there are very scarce published data on new technologies in dental care facilities. This is of very important, also given the increased risk of dental care personnel for contracting infectious diseases, including vaccine-preventable diseases such as hepatitis B, tuberculosis, and COVID-19, especially during droplet production and aerosol-generating procedures [10,11,12,13]. Indeed, a recent study using occupational data from the Canadian Occupational Information Network found that four dental occupations (dental hygienists and therapists, dental assistants, dentists, and denturists) ranked as the top four occupations with the highest exposure to workplace indicators that increase the risk of exposure to COVID-19 [14]. Nevertheless, there are gaps in our knowledge regarding the extent of spatiotemporal contamination, in relation to proximity to the operational site, duration, and complexity of dental treatment, and particularly in relation to viruses [15]. Moreover, a recent Cochrane meta-analysis of 16 studies conducted to assess the effectiveness of methods used to minimize aerosol production and reduce aerosol contamination during dental procedures found that all studies estimated bacterial contamination at various distances using colony-forming units (CFUs), while no study estimated the clinically important difference in CFU, transmission of infection, or viral contamination [16]. Overall, there was limited benefit found from particular interventions (e.g., high-volume evacuators and dental isolation combination systems); however, the researchers pointed out the complete absence of published evidence on ventilation, ionization, ozonation, UV light, and fogging [16]. Studies are needed to draw robust evidence for viral contamination in aerosols, as well as transmission of infection in dental practice.

Indoor air quality is also a critical issue of safety and quality in non-healthcare facilities. In modern societies, individuals spend more than 80% of their daily time indoors, mostly in working spaces, entertainment facilities, and residences. The turnover of attention over this specific subject resulted from reports from tenants of differing indoor areas, in which a variety of non-specific symptoms such as eye and neck irritation, headache, and deep breathing were reported [6]. In the following research, symptoms were reported to be related to low indoor air quality, and the term Sick Buildings Syndrome was adopted [17]. Until then, emphasis was placed on studying outdoor air quality in urban areas due to the environmental changes deriving from industries and automobile circulation [17]. Moreover, indoor air quality was indirectly correlated to outdoor air quality as well [17]. According to the International WELL Building Institute, the exposure to infective agents due to poor air quality may result in a series of health issues such as frequent headaches, asthma, high arterial blood pressure, infections, and even malignancies [18]. Therefore, addressing the quality of indoor air and its impact on humans’ physical and mental health is imperative for their well being.

The aim of this non-systemic review is to present the current state of knowledge on the nature and dynamics of air splatters and to provide an update to air quality management and control strategies in dental care facilities, implementing new technologies.

## 2. Methods

**Figure 1:**
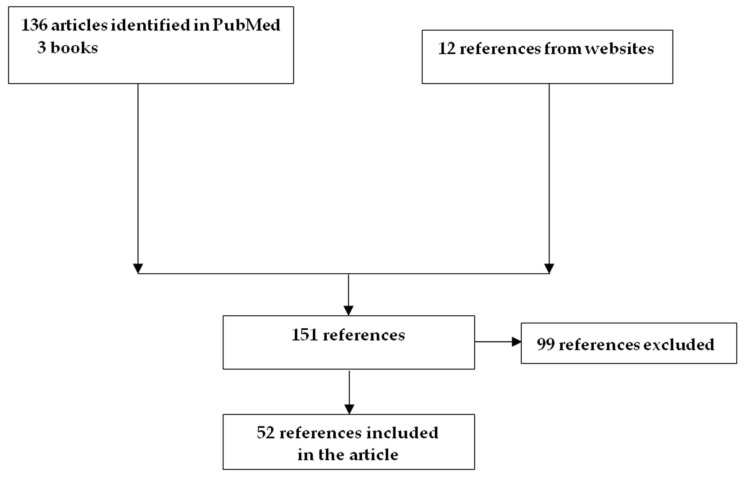
Flow diagram results of literature search.

This is a non-systemic review. Articles published in PubMed through 1 March 2022 were selected using combinations of the words “air splatters”, “dental handpieces aerosol”, “dental office indoor air quality”, “ultrasonic cleaners emission”, and “infection control”. We also used information from websites and books. We read the abstracts of 151 references and excluded 99 based on the fact that they concerned data from hospitals or non-healthcare-associated workplaces. Overall, we included a total of 52 references based on their relevance to the studied topic (Figure 1).

## 3. Characteristics of Air Splatters

Air splatters are liquid or solid particles floating in the air based on turbulent flow [18]. They may be visible, such as fog, but are more frequently invisible, when they are in the form of dirt or pollen. They are often divided into small drops, which are referred to as aerosol, and in larger drops are named droplets [19,20].

Large droplets of size 50–100 μm fall on the ground prior to their evaporation, causing spatial contamination [20]. Large droplets can contribute to the spread of pathogens and subsequently to infection through direct contact with a contaminated surface or when an infectious patient coughs, sneezes or talks intensely [20,21]. In contrast, because of the air splatters’ small size, levitation dynamics surpass gravity, permitting levitation in the air for longer time periods, or evaporation before reaching the floor, leaving solid (core) droplets free to float in extended distances, leading to airborne transmission [20,21].

Respiratory air splatters are created when the air transposes a liquid layer. However, multiple factors may have an impact on this process. Liquid layer viscosity is an essential determinant of air splatter production, since by increasing the surface tension of the overall droplet formation, the production of smaller droplets will potentially travel farther [22,23].

According to Morawska [23], droplets smaller than 100 μm, a size common in almost all droplets, evaporate before coming into contact with the floor. This means that droplets smaller than 100 μm can transmit the infection through the airway; however, a size of 5 μm has been also considered as a critical air droplet size [23]. It appears that air droplets can behave in any way depending on how fast they evaporate compared to how quickly they fall to the ground based on the atmospheric conditions of the room.

The latter argument questions how sufficiently air transferring and droplet spreading are distinguished. Some scientists consider the uncertainty of this activity, whereas others indicate that large droplets evaporate and turn into smaller droplets. In addition, they support that most of such activities create a vast variety of droplet sizes [24]. A plethora of epidemiologic studies claim that an infectious agent could be transferred merely through close contact, but such studies cannot efficiently differentiate between air splatter transfer of short distance and transfer through contact [25].

## 4. Procedures of Air Splatter Production

In healthcare settings, beyond the production of large droplets by patients, air splatters are mainly produced and spread through clinical examination, and diagnostic or therapeutic practices [20]. It is important to keep in mind that patients will produce their own air splatters, even when procedures that may produce air splatters are not performed. Such procedures may produce large and small sized air splatters [20]. Procedures that produce air splatters may cause spreading through different ways which are not usually used by microbes (e.g., a virus is typically transferred through contact or droplets). Such procedures may either directly produce air splatters or cause coughing or sneezing in the patient, a procedure which may be important when trying to temper the risk of transmission [20]. Despite the fact that respiratory secretions are the main source of air droplets, they might eventually be developed in different ways as well. In each surgical operation, air splatter pathogenic germs may be found in blood or tissues, such as human immunodeficiency virus (HIV), which has been traced in air splatters that were developed by surgical electric surgical tools [20]. Air splatters may also be produced by seemingly common objects, such as the fast-running tap water and basin coming from toilets [20]. Most protocols to prevent SARS-CoV-2 spread have emphasized the procedures of air splatter production [18]. However, it is of paramount significance to comprehend that such air splatters are also produced through the actions of people, including the one of simply breathing [23]. Virtually, every air mass passing through the respiratory tract will create droplets. The clinical significance depends on the number of droplets produced, their size, the concentration of infectious agents, the frequency with which the activity is performed, and the personal protective equipment (PPE) used by healthcare personnel. It should be noted that even though coughing produces a larger number of droplets of various sizes compared to a single breath, breathing is a continuing procedure leading to increased possibilities of producing more droplets in total. It is also worth mentioning that in case the majority of droplets produced through coughing might be small enough to linger in the air, the small size signifies that they add only a small fraction of the volume produced (potentially less than 0.1%), and consequently, only a small fraction of the total spreading of the virus [23]. Nonetheless, not every air droplet is full of viruses and even if it is, the load might not be enough to successfully transmit the infection. However, the smaller droplets can easily transmit the infection, despite carrying a smaller number of microorganisms [20].

Early studies concluded that most individuals mainly produce large droplets, which were found to be significantly limited due to the fact that the monitoring organs used were not sensitive to smaller sizes [24,25]. Recent studies indicate that 80–90% of particles produced by human breath are smaller than 1 μm in size. In spite of the questionability of the exact size of the droplets, the majority of research supports that speech, coughing, and sneezing produce droplets small enough to remain air transferred [22,26].

Interestingly enough, the total amount of air splatter produced varies greatly between individuals, with some people creating very little, while others acting as “superspreaders”. Vomiting, in which people can produce close to one million vomit virus particles, can also produce air splatters. Emesis produced by SARS patients was associated with the spread of disease in hospitals in Hong Kong in 2003, although it was not clear by which route, droplet contact or airborne, transmission occurred [23]. It is possible to track up to a million particles of viruses in each gram of waste and toilets, in which air splatters are known to exist. That form of air splatters is believed to have spread SARS in the building blocks of Amoy Garden in Hong Kong in 2003 [23]. However, whether such air splatters can transmit the infection still depends immensely on the amount produced, the concentration of the infected agents, the virulent ability of the germ, and environmental factors. In particular, the virus should be able to survive, either in the air or on a surface, until it enters a host [24]. The distance between patients is of paramount significance for infection transmission, since it is directly associated with the amount of inhaled droplets.

Mathematical models have been widely used to explore the precise role of various variables implicated in the transmission of infection. The size of droplet is a key characteristic. One droplet of 1000 μm will fall at a distance of 1 m in 0.3 s, while one droplet of 100 μm in size will need 3 s to fall 1 m away. In contrast, one droplet of 10 μm will need 300 s and one droplet of 1 μm will need 30,000 s. The time that a droplet remains in the air is a definite significant factor regarding how far away it may be transferred and how possible it is for healthcare personnel to be exposed [18,21,24].

Accurate size classifications are questionable, but Chen et al. proposed that the distribution of all droplets between 0.1 and 200 μm will be affected mainly from ventilation models and the initial speed of the droplet, despite gravity [26]. In other words, those droplets do not simply fall on the ground in 1–2 m away from the patient, as many practices of infection inspection suppose. However, the distribution of droplets is also impacted by several factors, including humidity, temperature, the model and the ventilation pattern, the initial speed, the group of individuals, and the size and the composition of core droplets [24,26]. Most of these factors are dynamic (shifts in droplet size due to evaporation and temperature change), rendering estimations difficult. In smaller sizes, Brown’s move, electric forces, thermical inclinations, and turbulent diffusion have major impacts [20]. Multiple calculations regarding the allocation of droplets have incorporated numerous hypotheses. For example, former studies made the assumption that the droplets enter the air without any speed, which is false, because coughing and sneezing may create substantial initial velocities of droplets [23]. It is estimated that droplets produced through ordinary breathing have a speed of approximately 1 m/s, through speaking a speed of approximately 5 m/s, through coughing a speed of 10 m/s, and through sneezing a speed of 20–50 m/s [24,27,28,29]. Thus, even if large particles are often supposed to land near the patient, this hypothesis is often false. A typical example is walking across the sea on a stormy day. Large droplets which often cross only a short distance, can effortlessly reach surfaces farther away than the shore [17].

Although mathematical models and scientific data greatly support the 2-m rule regarding ordinary breathing and talking, most experts suggest that coughing and sneezing contribute to the spreading of droplets significantly more [21,27,28]. However, this rule applies only to large droplets [27]. Smaller droplets remain captured in the air and consequently, they are able to cross longer distances [27]. Unfortunately, most of these models disregard the fact that patients usually cover their mouth and nose when sneezing. Therefore, sneezing in a healthcare facility or indoors in general will certainly change the allocation of the droplets and render the number of 7–8 m less likely [20].

Small droplets will remain in the air for much longer time periods (which will turn into air transferring cover), but the precise flight duration is unknown and may change significantly based on factors like temperature and humidity [18]. Through ordinary breathing, large droplets mainly fall on the ground within a radius of 2 m, but they may also evaporate and turn into small droplets. Coughing and sneezing may push those droplets much farther—at least 6 m [18]. It is worth mentioning that this allocation is probabilistic. There is no evidence promising that a droplet will stop before a certain distance. Occasionally, very small particles are not considered hazardous, because even if they can be inhaled, they remain in the air and are not retained in alveoli. However, this appears to be false, since 50% of those particles smaller than 1 μm will eventually return in the respiratory airways [20].

## 5. Air Splatters Walking Management/Control

Proper ventilation is a critical step for the management of bio-air splatter. Under ideal conditions, 65% of all air transferring droplets can be removed through air exchange; even if the air is not perfectly mixed [22], 20–60% of air droplets can be removed under realistic conditions [19]. In practice, each air exchange may remove half of all air splatters from a room. It is also recommended to disinfect the air using numerous systems, such as High Efficiency Particulate Absorbance (HEPA) filters and UV light; however, the effectiveness of portable air cleaners with HEPA filters to limit the air transferring spread of SARS-CoV-2 in the corridors of hospital units has not been recorded. The most essential mechanism for the air splatters management is the use of PPE, including the use of mask respirators N95 or KN95 [2,19]. It has been largely considered that as long as healthcare personnel are 2 m away from the patient, there is no risk from droplets [19]. However, this statement often is used as a principle without any reference, while there is evidence questioning its correctness. The idea that all large droplets will fall onto the floor within 2 m seems to have been suggested based on simple calculations, with hypotheses that have been questioned and with limited experiential data [27]. Unfortunately, as previously stated, most of the already existing data appear to contradict this hypothesis. For example, in a recent study with five volunteers, after mouth washing with food pigment/coloring, there was visible macroscopic contamination in four out of five participants beyond a 2 m distance, while simple pictures of sneezing in a dark field show a droplet cloud up to 8 m [27,29]. Moreover, a recent simulated dental experiment using fluorescein dye, revealed that unmitigated dental procedures had the potential to contaminate distant sites; however, almost all settled aerosol is detected within 10 min [30]. In another study, the highest concentration of influenza virus RNA copies in respirable particles during the hospitalization of a patient with influenza was recorded outside the room of the patient [31]. Therefore, we should not consider the 2 m rule to be absolutely safe. Furthermore, since the droplets spread in 3 dimensions, the concentration of the droplet is exponentially reduced as the healthcare personnel stays away from the patient. Additionally, data indicate that most droplets that are created from ordinary breathing fall within 1 m, even if coughing and sneezing significantly augment this allocation [29]. Generally, the more we stay away from the patient, the safer we are. In other words, it is more likely to become infected within a 50 cm distance than within 1 m. The risk still exists within a two 2 m distance, and it does not fall to 0. We are even safer at 4 or 8 m away from the patient (or even better, behind a closed door), this fact however cannot apply to the practice of dentistry.

In practice, this means that PPE must be removed (doffing) further as much as possible from the patient. In an ideal world, we would remove the PPE back from a curtain or a door, to completely limit infection from droplets. However, even if the distance is increased in order to eliminate the risk from droplets, in reality, it increases the risk of spreading the infection through contact in surfaces and fomites. Obviously, being in a clean corridor or space with contaminated PPE is not an ideal option. The risk of spreading infectious agents through contact with fomites is almost certainly higher than the risk of droplets in a distance longer than 2 m away from the patient. Hence, the 2 m rule is often practically effective, despite its lack of scientific accuracy. A study examined this subject and found evidence that droplets spread more than 2 m away in 8 out of 10 cases [32]. The authors concluded that their findings should be carefully interpreted. The authors also stated that the spatial limit of 1 m that is recommended for droplet precautions for the staff in public entrances (e.g., harbors, airports, railroad stations) does not rely on current scientific evidence [32].

## 6. Air Splatter Technical Management

Indoor air management in dental clinics is a critical and multifactorial issue. The ideal management of air quality automatically should aim to commit a large amount of polluting factors. It is estimated that infective and toxic factors primarily affect the respiratory system [33]. Restorative dentistry implements high velocity handpieces with water blast, multiple air–water syringes, and even the exploitation of different microparticles emission for the conservative treatment of periodontal disease or for the preventive cleaning of dental implants [34]. Such droplets are usually calculated between 0.5 and 5 μm in diameter and, depending on the relative humidity of the space, they may remain suspended, immobilized upon surfaces for hours [34]. It is possible that pathogenic agents, bacteria, and viruses that are included in those microdroplets might be inhaled and cause infection to the dental staff and even to patients. In a series of papers published in the *Infection Control Today* magazine, it was referenced that human coronavirus (HCoV-)229E may remain infective on certain surfaces for at least 2 h to 9 days [35]. Following the evaluation of a number of disinfectant solutions suitable for surfaces, researchers agreed on the important effectiveness of alcoholic solutions with alcohol content of 62–71%; however, others such as benzalkonium chloride and 0.55% orthopthalaldehyde were less effective [35]. As early as in 2010, the United States Centers for Disease Control and Prevention supports the usage of rubber dams where possible, alongside with the urge for high aspiration suction. However, considering that dental procedures may be executed without dental chair side assistant support, the urge and effectiveness of severe intake may vary. Despite the absence of international guidelines regarding the presence of aerosols in dental clinics, it is generally recommended that suspended particles transferring bacteria should not exceed the limit of 10 particles per m^3^ [36]. It is of interest though, that such levels may be accomplished at a dental office in multiple phases of its operation. In a clinical research article involving odontostomatological examination (screening), cavity preparations assisted by high velocity handpieces, ultrasonic supported periodontal treatment, and tooth extraction, air splatters were produced and included, among others, staphylococci and micrococci, with and without the support from an Air Cleaning System (ACS) [36,37]. Regardless, we may conclude that the used ACS was effective in the reduction in bacterial air splatters. Although the usage of an ACS to reduce air splatters may not be required in every installment, this equipment has been shown in general to provide a safer working environment, for the patients as well as for the dental staff. Allergenics and toxic agents are suspended in the area of the dental office, including essential oils, polymeric materials, organic solvents, aldehydes, different catalysts, and microparticles of different sizes containing mercury, pollen, bacteria, viruses, volatile organic compounds (VOCs), and carbon dioxide, products of combustion primary from human action [37]. Due to the frequency of airborne infections in the last 15 years, multiple air cleaning devices have been designed, produced, and tested, along with special aspiration catching, inactivating, and infiltrating microparticles [37]. In the past decade, different portable or wheeled devices have been suggested that they can clean the air at the dental office and simultaneously remove pathogenic microorganisms [36,37]. HEPA filters should be used to ensure an efficient air cleaning device with the capacity to restrain microparticles and allergens. Those devices were recently embellished with UV radiation production bulbs or with plasma devices, inducing complementary cleaning of the already filtrated air [37]. Many of these devices are designed to monitor the air of general venue in which the dental unit is installed, while others are equipped with a special suction extension of 10–20 cm in diameter, reaching the work scope and in particular, the patient’s mouth. All those devices are space-possessive, and they approach the patient from the assistant staff area or the areas occupied by the cuspidor and suction systems [38,39,40]. In a recent publication, the air quality in indoor areas of the University of Athens School of Dentistry (Athens, Greece) was continuously recorded for several weeks during the COVID-19 pandemic, before and after the placement of air purification devices [41]. In this real-life study, a significant reduction in the pollutants configurated as microparticles, VOCs, and carbon dioxide was noticed, when the air purification devices were operating for at least 10 h during the presence of students, professors, supporting staff, and patients [41]. In particular, levels of particular matter 10 (PM_10_) and PM_2.5_ remained significantly low during almost the entire experimental period below the daily standard exposure limit of 25 μg/m^3^ proposed by the World Health Organization [41]. Levels of total VOCs were also low [41]. In addition, a randomized clinical trial recently evaluated the efficacy of an air purifier device with HEPA 14 filter in reducing the number of suspended particles generated during dental healthcare services as a vector of COVID-19 [42]. The latter study reported an 83% higher efficacy in the intervention group compared with the control group, while the contamination from a microbiological point was decreased by 69–80% [42].

Suction systems are utterly necessary. Ideally, the installment of the electric motor in a secure underground area releasing infected air in outdoor environments should be chose. If not, released air should be captured through specialized filtration eliminating the recycling and diffusion of pathogenic microorganisms [41]. It is worth evaluating the need for reestablishing dental units to reclaim space, through the restricting and/or abolition of the cuspidor and the incorporation of a specialized suction unit in the entire system. According to Tsoi et al. [43], during surgical procedures, an external oral suction or high-volume suction should be used in conjunction with low-volume suction, in order to reduce the spread of aerosols and droplets in a dental clinic environment [43].

The diameter of the hose of the surgical suctions currently is about 14 mm, small enough to capture the emitted air splatter, resulting in mist exiting from the mouth [41]. Ideally, intraoral suction of aerosols should be in close proximity with the field of collision of the air/water beam or air/water/microparticles in case of air powder abrasive systems or airflows. With the development and frequent utilization of laser technology, the need for re-design of units arises [41]. On the contrary, large air purifiers, parallel to the presence of multiple HEPA filters, contain a powerful electric motor, capable of aspirating up to 600 cubic meters per hour, and a widespread inlet with a diameter of 14 cm, which can approach the working scope with great proximity [38]. The placement of the orifice in position 4 clockwise near the patient, allows the aspiration of a large amount of emissions and at the same time, the assistant who undertakes the collection of primary emission of air splatters is not hindered [41]. The presence of air renewal systems in hospital clinical areas and dental offices, especially in those where more than one dental unit coexists, is of major importance.

**Figure 2:**
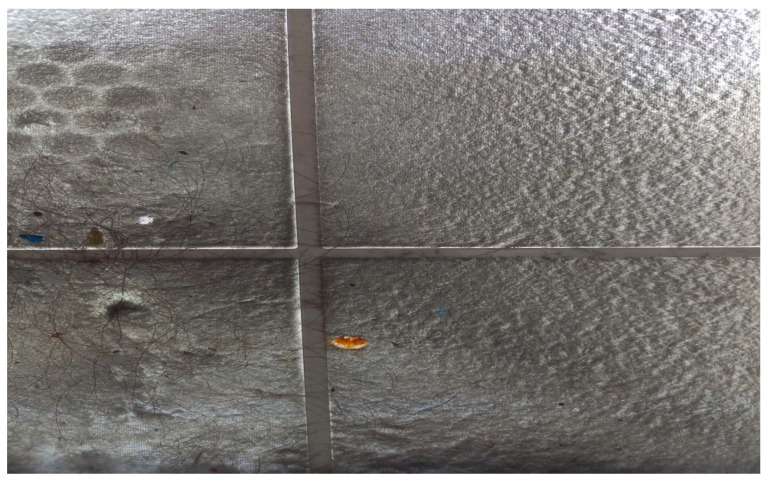
Close view of the sieve from the air purifier (School of Dentistry, National and Kapodistrian University of Athens).

It is highly recommended to frequently inspect, clean, decontaminate the ordinary sieves, and replace both the carbon and the HEPA filters, according to the manufacturers’ guidelines [38]. In case the sieves cleaning is neglected, a velvety cloth-like mass is observed which, together with various macroparticles, microparticles and bristles, blocks the filter and prevents the air that is drawn in from being cleaned by the next specialized filters (Figure 2).

**Figure 3:**
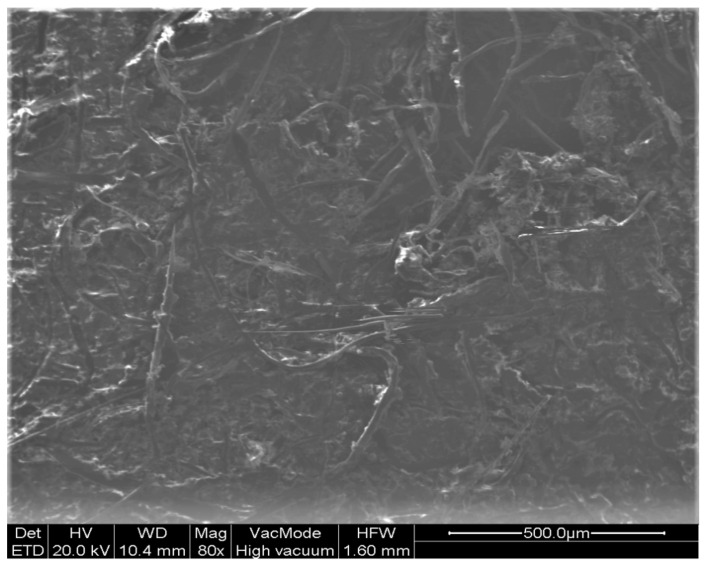
Scanning Electron Microscopy picture of the surface of the deposition (SEI 80×) (School of Dentistry, National and Kapodistrian University of Athens).

**Figure 4:**
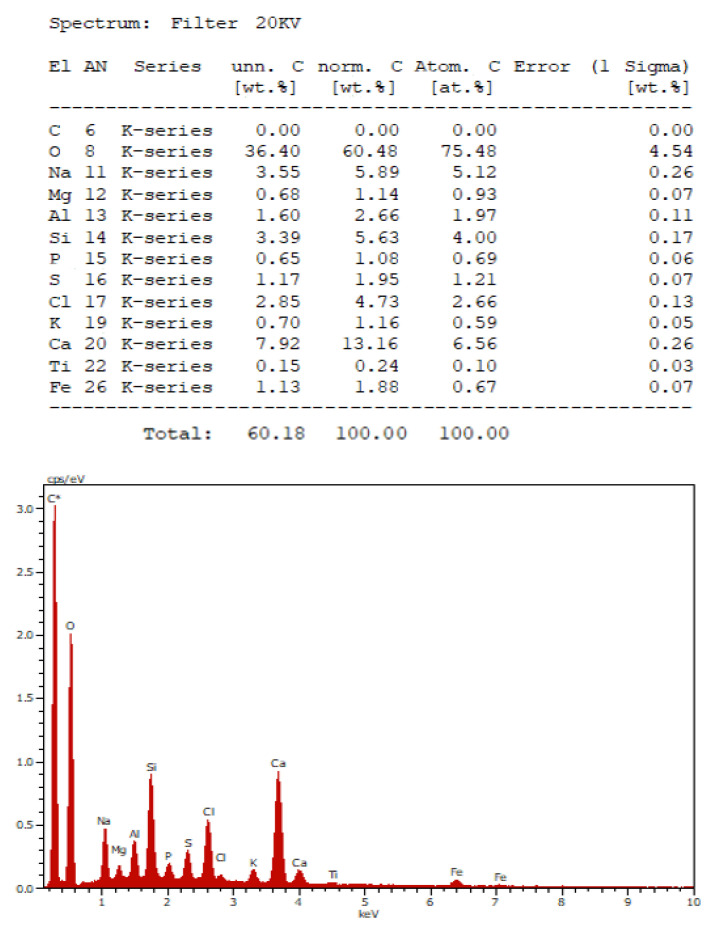
Energy dispersive X-ray analysis of the depositions (School of Dentistry, National and Kapodistrian University of Athens).

This mass contains a wide range of elements such as sodium, calcium, silicon, chlorine, iron, and carbon compounds with oxygen (Figure 3 and Figure 4).

Lately, a new therapeutic protocol for the removal of bacterial biofilm, as well as for the prevention and therapy of different periodontal and peri-implant diseases and conditions has been introduced in daily dental practice [44]. This method is named Guided Biofilm Therapy and uses air-abrasive decontamination technology as well as ultrasonic removal of hard microbial deposits. For this purpose, three new handpieces have been invented by Electron Mechanical Systems (EMS, Nyon Suisse), two for air-abrasive decontamination (AirFlow, PerioFlow) and one for ultrasonic debridement (Piezon-NoPain). The device that supplies these handpieces delivers compressed air at a supply of 450–700 kPa and water at a supply of 200–500 kPa. As a result, the Airflow handpiece has a power jet that reaches 400 km/h, providing a uniform spray pattern, whereas the Perioflow handpiece, used for probing depths equal or greater than 4 mm, has a trilateral powder outlet, which means that it sprays the mix of powder and water towards three different directions, since its effectiveness is based on a vortex principle. Another important characteristic is the size of the particles that constitute the powders used either supra- or subgingivally. Glycine, which is issued for supragingival application, has a particle size of 40 μm and 75.07 g/mol molecular weight, whereas erythritol, which is the only one indicated for subgingival use, has a particle size of 14 μm and 122.12 g/mol molecular weight which explains its efficacy [45].

**Figure 5:**
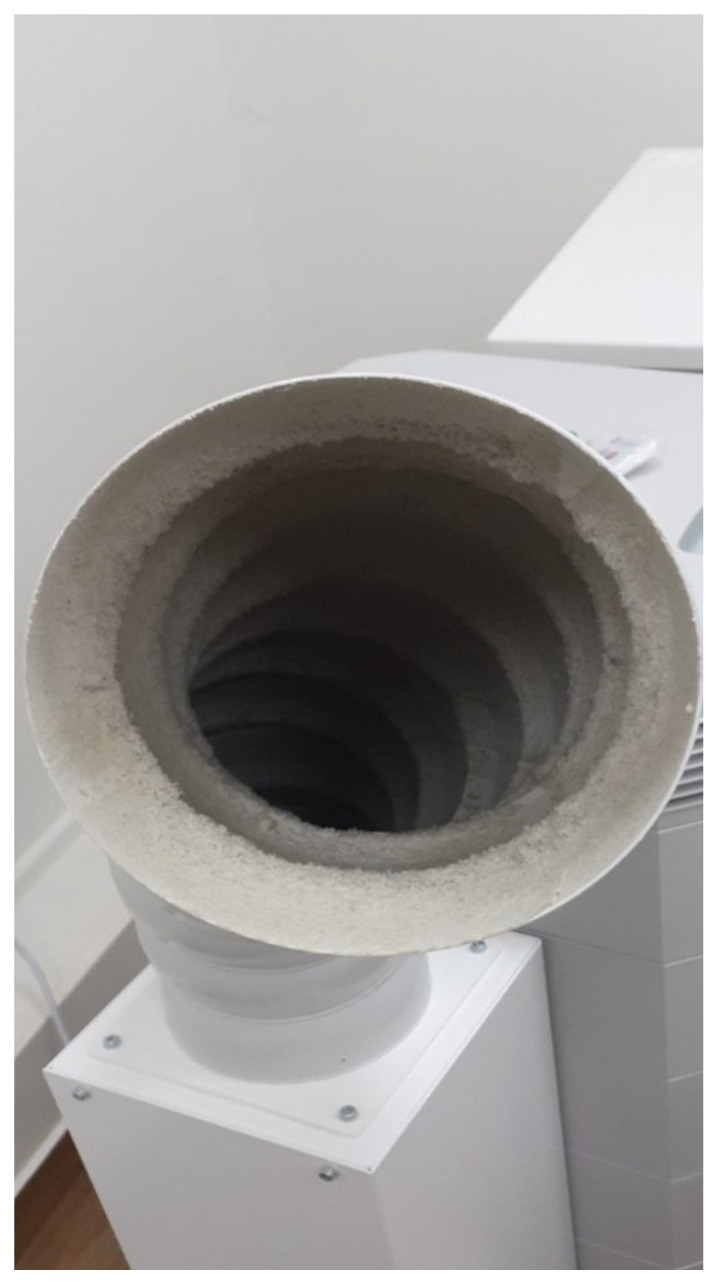
Hose contaminated from microparticles (School of Dentistry, National and Kapodistrian University of Athens).

**Figure 6:**
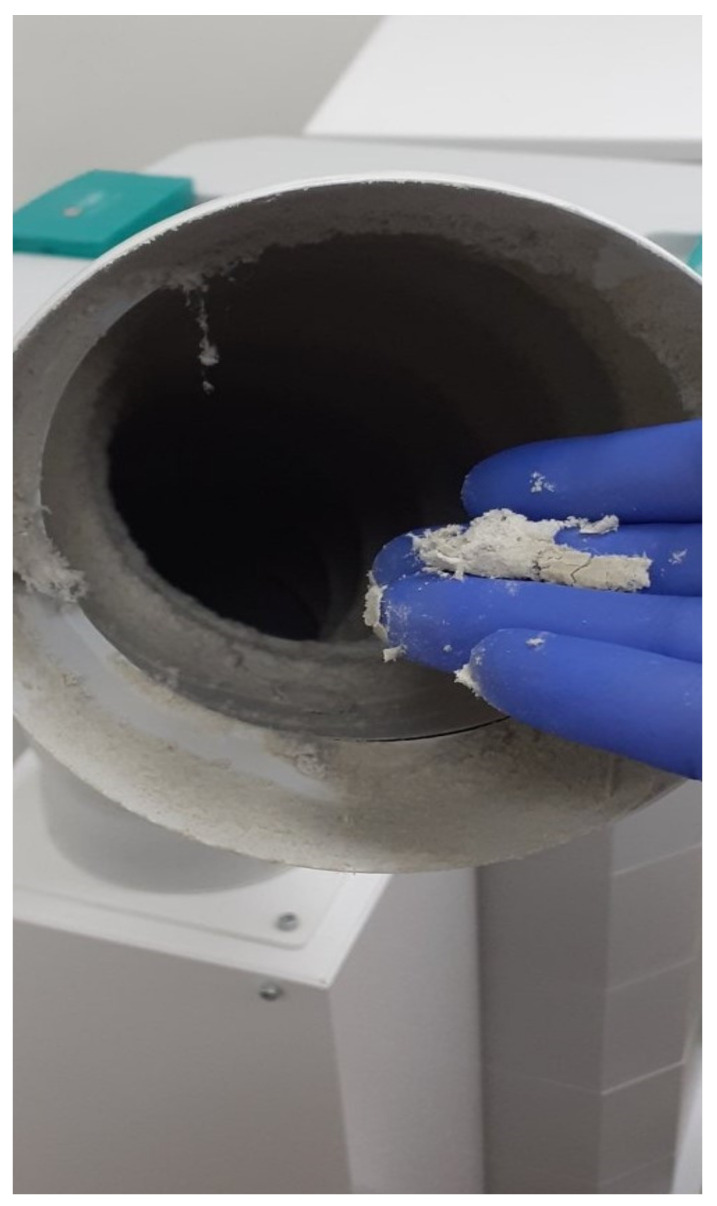
Close view of the previous picture (School of Dentistry, National and Kapodistrian University of Athens).

The device provides the clinician the opportunity to alter the water supply and power for each handpiece. The manufacturing company recommends, depending on the severity of the disease or the target (stain or biofilm removal, scaling and root planning, etc.), 30–100% power supply and 70–100% water supply for the ultrasonic handpiece, whereas 30–100% power supply for supragingival use or 30–60% power supply for subgingival use and 100% water supply for the Airflow and Perioflow systems. However, in order to minimize aerosol, the handpieces utilizing air-abrasion must be used at maximum power, but preferably at 50%, for both supra- and subgingival application, and indeed, at 100% water supply, so as to restrict powder dispersion as much as possible (Figure 5 and Figure 6).

Recently, a new nozzle for the Airflow has been introduced to the market that sprays half the powder quantity, compared to the former. Although the most effective tips for ultrasonic scalers are the thinnest, it is well known that the thinner the tip, the greater the oscillation at the last millimeters of the working tip and, therefore, the greater the dispersion of water droplets, especially when high powers are necessary [46].

The presence of air intake and aspiration facilities to and from the external environment, equipped with suitable filters and selective speed is the most ideal technique to ensure quality and safe operating conditions in dental care facilities, especially those that produce air splatters. Frequent disinfection is also crucial, as well as the coverage of the surfaces peripherally of the patient and the staff. Effective cleaning of the floors of the operative area, with appropriate cleaning materials at frequent intervals, is also needed in order to limit the diffusion of pathogenic microorganisms.

## 7. Utilization of UV Radiation for Air and Surface Sterilization

The environment may contribute to transmission of infectious agents in dental care facilities. A multi-center microbiological environmental investigation conducted in six dental clinics across Italy revealed high microbial contamination of water, air, and surfaces, mainly during dental treatments [47]. Following the production of electric bulbs emitting UV radiation with significantly prolonged life spans, this technology was used for processing purified water, as well as sewage disposal, air sterilization, and sterilization of many objects such as toothbrushes and computer accessories. The UV moves on the right side of the spectrum approximately between 10 and 40 nm. The germicidal range of UV radiation is within the length waves of 100–280 nm, known as UV-C with the largest length wave for germicidal action being 265 nm. This spectrum area of UV light is absorbed from the DNA and RNA of microorganisms, causing changes in their structure, rendering the microorganisms incapable of reproducing [48]. In case of using UV bulbs for the sterilization of surfaces and areas, it is necessary to achieve the highest germicide/virucide UV radiation, where 90% of this energy is produced at 254 nm. This radiation is very close to the summit of the curve between germicidal effectiveness of 265 nm, which is now the deadliest length of wave for microorganisms due to the fact that all viruses contain RNA or DNA, and they are therefore prone to radiation [45]. Currently, there is accumulated information regarding the appropriate dosage for the deactivation of different microorganisms. Bacteria are more easily deactivated than viruses, whereas fungi and spores are even harder to deactivate through UV radiation [45]. For the above mentioned reasons, sterilization in the autoclave through liquid heat in the form of pressurized moist heat (steam) remains the safe gold standard method. It is important to highlight that UV radiation is categorized as a decontamination technique, consisting of meticulous cleaning and sterilization [48,49,50].

There are technical restrictions in using UV radiation technology [44]. UV radiation functions in a transparent way through ranges, solely radiating surfaces in direct exposure, and consequently other surfaces which insert into the route and are referred to as “shadow areas” [44]. Shadow areas do not absorb adequate luminescence for sterilization, since UV fades away by relocating the light source of UV radiation to another position [49,50].

Distancing plays an essential role in the effectiveness of UV light. The light power UV-C is reduced as it is fended off from the light source, following the law of reversed square. This means that in double distance, UV-C will have one fourth of the power compared to the initial reference point [49]. This comparison determines the effectiveness of only one UV light source to provide adequate sterilization. Most systems focus on quantifying the output UV-C in a granted distance and utilizing this distance to determine the time of exposure so that it is effective [49,50].

The UV light does not properly penetrate organic materials and visible organic waste. Therefore, for optimal results, it is necessary that UV-C is used after the ordinary cleaning of the area, to certainly remove any organic material from surfaces [48,49,50]. UV sterilization devices are found to be flawed in terms of the extensive production of ozone, causing irritation to the respiratory system and provoking intense irritation to asthma patients. However, UV restricts the need for utilizing technical spray, which requires evaluation or creating a model of air flow through UV-C as it occurs in fog systems. Additionally, the UV technique does not need to isolate areas, which facilitates its implication. Moreover, the amounts of time for sterilization last for approximately 15 min [48]. This allows incredibly fast records of operating for dental care areas. Due to its simplicity, UV-C sterilization is incredibly easy to comprehend and prepare. All surfaces in a certain distance will succeed a specific level of sterilization in a particular amount of time, under the condition that light is not hindered from being exposed to that surface. This technique is being successfully implemented in operation areas and medical offices, including dental care facilities, patients’ rooms, and ambulances, but also public transportation and areas of processing and conserving food supplies among others [48]. Recently, UV tubes have been incorporated in air purifiers, complementarily processing the already purified air deriving from the air passage of environment from microcrystalline carbon filters and HEPA filters [50]. According to national and international authorities on air splatter research and management, the preservation of air quality of indoor areas is critical for limiting the spread of airborne diseases in working areas, including dental care facilities [51,52].

## 8. Conclusions

The identification of the best practices to maximize safety for patients and healthcare personnel, including dental staff in dental care facilities, is imperative. This is also crucial given the risk of exposure to several infectious diseases, including vaccine-preventable diseases such as tuberculosis and COVID-19 in the context of droplet production and aerosol-generating procedures [10,11,12,13,14]. Review of published evidence regarding the dynamics of air splatters in healthcare facilities indicates areas of uncertainty. Considering the lack of standardized data, attention should be paid not only to the search for evidence indicating that a practice is harmful, but also that a practice is safe enough to be implemented. It is clinically insignificant to distinguish the emissions in air transferring air splatters and large droplets, given that larger droplets may become smaller as they evaporate. At the same time, there is increasing evidence that viruses can be transmitted from patients to a larger extent than what current models predict. The current literature also indicates that almost any respiratory activity, including ordinary breathing, can create air splatters. However, the hazards of air splatters are much less than the risk of droplets and the risk of close contact with an infectious patient. The utilization of air purifiers are highly recommended in dental healthcare facilities to mitigate spread of infection. Their operation should be continuous, not only during but also after the treatment of the patients. Cleaning and maintenance of the purifier sieves and filters should be scheduled, while the frequent renovation of the indoor air through physical ventilation, by means of open wondows, is of critical importance. More research on environmental contamination and particularly on viral contamination conducted under real dental care conditions is needed to guide infection prevention interventions. Healthcare units and particularly dental clinics where air splatters are commonly produced should ensure that healthcare personnel are not infected during their work, while providing exemplary and safe care to their patients.

## References

[1] Morgenstern J. Aerosols, Droplets, and Airborne Spread. Everything you Could Possibly Want to Know. https://first10em.com/aerosols-droplets-and-airborne-spread/.

[2] Maltezou H.C., Tseroni M., Vorou R., Koutsolioutsou A., Antoniadou M., Tzoutzas I., Panis V., Tzermpos F., Madianos P. (2021). Preparing dental schools to refunction safely during the COVID-19 pandemic: An infection prevention and control perspective. J. Infect. Dev. Ctries.

[3] Li Y., Qian H., Hang J., Chen X., Cheng P., Ling H., Wang S., Liang P., Li J., Xiao S. (2021). Probable airborne transmission of SARS-CoV-2 in a poorly ventilated restaurant. Build Environ..

[4] Cheng V.C.C., Fung K.S.C., Siu G.K.H., Wong S.C., Cheng L.S.K., Wong M.S., Lee L.K., Chan W.M., Chau K.Y., Leung J.S.L. (2021). Nosocomial outbreak of COVID-19 by possible airborne transmission leading to a superspreading event. Clin. Infect. Dis..

[5] Miller C. (2016). Infection Control and Management of Hazardous Materials for the Dental Team.

[6] Wood P.R. (1992). Cross Infection Control in Dentistry, a Practical Illustrated Guide.

[7] Masia M.D., Dettori M., Deriu G.M., Soddu S., Deriu M., Arghittu A., Azara A., Castiglia P. (2021). Microbial monitoring as a tool for preventing infectious risk in the operating room: Results of 10 years of activity. Atmosphere.

[8] Centers for Disease Control and Prevention (CDC) (2003). Guidelines for Environmental Infection Control in Health-Care Facilities—Recommendation of CDC and the Healthcare Infection Control Practices Advisory Committee (HICPAC).

[9] Helmis C.G., Tzoutzas I., Flocas H.A., Halios C.H., Stathopoulou O.I., Assimakopoulos V.D., Panis V., Apostolatou M., Sgouros G., Adam E. (2007). Indoor air quality in a dentistry clinic. Sci. Total Environ..

[10] Jungo S., Moreau N., Mazevet M.E., Ejeil A.L., Duplan M.B., Salmon B., Smail-Faugeron V. (2021). Prevalence and risk indicators of first-wave COVID-19 among oral health-care workers: A French epidemiological survey. PLoS ONE.

[11] Chavis S.E., Hines S.E., Dyalram D., Cole Wilken N., Dalby R.N. (2021). Can extraoral suction units minimize droplet spatter during a simulated dental procedure?. J. Am. Dent. Assoc..

[12] Petti S. (2016). Tuberculosis: Occupational risk among dental healthcare workers and risk for infection among dental patients. A meta-narrative review. J. Dent..

[13] Malsam R., Nienhaus A. (2021). Occupational infections among dental health workers in Germany-14-year time trends. Int. J. Environ. Res. Public Health.

[14] Singhal S., Warren C., Hobin E., Smith B. (2021). How often are dental care workers exposed to occupational characteristics that put them at higher risk of exposure and transmission of COVID-19? A comparative analysis. J. Can. Dent. Assoc..

[15] Gallagher J.E., Sukriti K.C., Johnson I.G., Al-Yaseen W., Jones R., McGregor S., Robertson M., Harris R., Innes N., Wade W.G. (2020). A systematic review of contamination (aerosol, splatter and droplet generation) associated with oral surgery and its relevance to COVID-19. BDJ Open.

[16] Nagraj S.K., Eachempati P., Paisi M., Nasser M., Sivaramakrishnan G., Verbeek J.H. (2020). Interventions to reduce contaminated aerosols produced during dental procedures for preventing infectious diseases. Cochrane Database Syst. Rev..

[17] Jones A.P. (1999). Indoor air quality and health. Atmos. Environ..

[18] International WELL Building Institute Air. https://standard.wellcertified.com/air.

[19] Tellier R. (2009). Aerosol transmission of influenza A virus: A review of new studies. J. R. Soc. Interface.

[20] Judson S.D., Munster V.J. (2019). Nosocomial transmission of emerging viruses via aerosol-generating medical procedures. Viruses.

[21] Nicas M., Nazaroff W.W., Hubbard A. (2005). Toward understanding the risk of secondary airborne infection: Emission of respirable pathogens. J. Occup. Environ. Hyg..

[22] Fiegel J., Clarke R., Edwards D.A. (2006). Airborne infectious disease and the suppression of pulmonary bioaerosols. Drug Discov. Today.

[23] Morawska L. (2006). Droplet fate in indoor environments, or can we prevent the spread of infection?. Indoor Air.

[24] Xie X., Li Y., Chwang A.T., Ho P.L., Seto W.H. (2007). How far droplets can move in indoor environments revisiting the Wells evaporation-falling curve. Indoor Air.

[25] Hinds W.C., Cottone J.A., Terezhalmy G.T., Molinari J.A. (1982). Practical Infection. Aerosol Technology Properties, Behavior, and Measurement of Airborne Particles.

[26] Chen W.Q., Ling W.H., Lu C.Y., Hao Y.T., Lin Z.N., Ling L., Huang J., Li G., Yan G.M. (2009). Which preventive measures might protect health care workers from SARS?. BMC Public Health.

[27] Asadi S., Wexler A.S., Cappa C.D., Barreda S., Bouvier N.M., Ristenpart W.D. (2019). Aerosol emission and super emission during human speech increase with voice loudness. Sci. Rep..

[28] Noti J.D., Blachere F.M., McMillen C.M., Lindsley William G., Kashon M.L., Slaughter D.R., Beezhold D.H. (2013). High humidity leads to loss of infectious influenza virus from simulated coughs. PLoS ONE.

[29] Papineni R.S., Rosenthal F.S. (1997). The size distribution of droplets in the exhaled breath of healthy human subjects. J. Aerosol. Med..

[30] Holliday R., Allison J.R., Currie C.C., Edwards D.C., Bowes C., Pickering K., Reay S., Durham J., Lumb J., Rostami N. (2021). Evaluating contaminated dental aerosol and splatter in an open plan clinic environment: Implications for the COVID-19 pandemic. J. Dent..

[31] Bourouiba L. (2020). Turbulent gas clouds and respiratory pathogen emissions: Potential implications for reducing transmission of COVID-19. JAMA.

[32] Hui D.S., Chan M.T., Chow B. (2014). Aerosol dispersion during various respiratory therapies: A risk assessment model of nosocomial infection to health care workers. Hong Kong Med. J..

[33] Loh N.W., Tan Y., Taculod J., Gorospe B., Teope A.S., Somani J., Tan A.Y.H. (2020). The impact of high-flow nasal cannula (HFNC) on coughing distance: Implications on its use during the novel coronavirus disease outbreak. Can. J. Anaesth..

[34] Cummings K.J., Martin S.B., Lindsley W.G., Othmpangat S., Blachere F.M., Noti J.D., Beezhold D.H., Roidad N., Parker J.E., Weissman D.N. (2014). Exposure to influenza virus aerosols in the hospital setting: Is routine patient care an aerosol generating procedure?. J. Infect. Dis..

[35] Bahl P., Doolan C., de Silva C., Chughtai A.A., Bourouiba L., MacIntyre C.R. (2020). Airborne or droplet precautions for healthworkers treating COVID-19?. J. Infect. Dis..

[36] Joshi S.M. (2008). The sick building syndrome. Indian J. Occup. Environ. Med..

[37] Steiner C., Let Airflow Show Pathogens the Door (or Window or Vent) Infection Control Today. https://www.infectioncontroltoday.com/view/let-airflow-show-pathogens-the-door-or-window-or-vent-.

[38] Diamond F., Best Approach to Disinfecting Surfaces Amid Novel Coronavirus Outbreak Infection Control Today. https://www.infectioncontroltoday.com/view/best-approach-disinfecting-surfaces-amid-novel-coronavirus-outbreak.

[39] Whyte W., Green G., Whyte W.M. (2012). Removal of microbe carrying particles by high-efficiency air filters in clean rooms. Intern. J. Vent..

[40] Whyte W., Agricola K., Dercks M. (2015). Airborne Particle Deposition in Clean Rooms. Deposition Mechanisms. Clean Air Contain. Rev..

[41] Tzoutzas I., Maltezou H.C., Barbaressos N., Tasios P., Efthymiou C., Assimakopoulos M.N., Tseroni M., Vorou R., Tzermpos F., Antoniadou M. (2021). Indoor air quality evaluation using mechanical ventilation and portable air purifiers in an academic dentistry clinic during the COVID-19 pandemic in Greece. Int. J. Environ. Res. Public Health.

[42] Capparè P., D’Ambrosio R., De Cunto R., Darvizeh A., Nagni M., Gherlone E. (2022). The usage of an air purifier device with HEPA 14 filter during dental procedures in COVID-19 pandemic: A randomized clinical trial. Int. J. Environ. Res. Public Health.

[43] Tsoi J.K.H., Ding H., Hon K., Leung Y.Y. (2021). The spread of droplets and aerosols of surgical motor handpiece irrigation using different suction systems. Front. Dent. Med..

[44] EMS Technical Properties Airflow Prophylaxis Master. https://www.ems-dental.com/el/products/airflow-prophylaxis-master.

[45] EMS Treatment Recommendations Guided Biofilm Therapy. ems-dental.com.

[46] Vyas N., Pecheva E., Dehghani H., Sammons R.L., Wang Q.X., Leppinen D.M., Walmsley A.D. (2016). High speed imaging of cavitation around dental ultrasonic scaler tips. PLoS ONE.

[47] Pasquarella C., Veronesi l., Castiglia P., Liguori G., Montagna M.T., Napoli C., Rizzetto R., Torre I., Masia M.D., Di Onofrio V. (2010). Italian multicentre study on microbial environmental contamination in dental clinics: A pilot study. Sci. Total Environ..

[48] Cumbo E., Gallina G., Messina P., Scardina G.A. (2020). Alternative methods of sterilization in dental practices against COVID-19. Int. J. Environ. Res. Public Health.

[49] Advanced Biotechnologies Inc Is UV Sterilization Effective for Viruses and Bacteria?. https://abionline.com/is-uv-sterilization-effective-for-viruses-and-bacteria/.

[50] Rensair Clean Air for Every Space. https://rensair.com.

[51] Abu-Hammad O., Alnazzawi A., Babkair H., Jambi S., Mirah M., Abdouh I., Aljohani R.S., Ayeq R., Ghazi L., Al-Subhi H. (2021). COVID-19 infection in academic dental hospital personnel: A cross-sectional survey in Saudi Arabia. Int. J. Environ. Res. Public Health.

[52] Hellenic Society for Research on Air Splatters The Significance of Indoor Air Quality in Controlling COVID-19. https://youtu.be/fFdDM_33bVE.

